# Barratt Impulsivity and Neural Regulation of Physiological Arousal

**DOI:** 10.1371/journal.pone.0129139

**Published:** 2015-06-16

**Authors:** Sheng Zhang, Sien Hu, Jianping Hu, Po-Lun Wu, Herta H. Chao, Chiang-shan R. Li

**Affiliations:** 1 Department of Psychiatry, Yale University, New Haven, CT 06519, United States of America; 2 Department of Psychology, South China Normal University, Guangzhou, China; 3 Department of Psychiatry, China Medical University, Tai-Chung, Taiwan; 4 Department of Medicine, Yale University, New Haven, CT 06519, United States of America; 5 Department of Medicine, VA Connecticut Healthcare Systems, West Haven, CT 06516, United States of America; 6 Interdepartmental Neuroscience Program, Yale University, New Haven, CT 06520, United States of America; 7 Department of Neurobiology, Yale University, New Haven, CT 06520, United States of America; Central Institute of Mental Health, GERMANY

## Abstract

**Background:**

Theories of personality have posited an increased arousal response to external stimulation in impulsive individuals. However, there is a dearth of studies addressing the neural basis of this association.

**Methods:**

We recorded skin conductance in 26 individuals who were assessed with Barratt Impulsivity Scale (BIS-11) and performed a stop signal task during functional magnetic resonance imaging. Imaging data were processed and modeled with Statistical Parametric Mapping. We used linear regressions to examine correlations between impulsivity and skin conductance response (SCR) to salient events, identify the neural substrates of arousal regulation, and examine the relationship between the regulatory mechanism and impulsivity.

**Results:**

Across subjects, higher impulsivity is associated with greater SCR to stop trials. Activity of the ventromedial prefrontal cortex (vmPFC) negatively correlated to and Granger caused skin conductance time course. Furthermore, higher impulsivity is associated with a lesser strength of Granger causality of vmPFC activity on skin conductance, consistent with diminished control of physiological arousal to external stimulation. When men (n = 14) and women (n = 12) were examined separately, however, there was evidence suggesting association between impulsivity and vmPFC regulation of arousal only in women.

**Conclusions:**

Together, these findings confirmed the link between Barratt impulsivity and heightened arousal to salient stimuli in both genders and suggested the neural bases of altered regulation of arousal in impulsive women. More research is needed to explore the neural processes of arousal regulation in impulsive individuals and in clinical conditions that implicate poor impulse control.

## Introduction

Characterized by behavioral disinhibition and rash actions, trait impulsivity may dispose individuals to negative consequences. An important dimension of trait impulsivity concerns the regulation of physiological arousal. A number of leading theories of personality posit that impulsivity–as a component trait of extraversion–is characterized by under-arousal at rest and greater increases in arousal in response to stimulation [1–5]. Impulsive individuals seek stimulation to maintain an optimal level of arousal.

In support, many studies demonstrated increased physiological arousal to stimulation in individuals higher on impulsivity. For instance, impulsivity in association with risk taking behavior was correlated to increased skin conductance reactivity to arousing stimuli [6]. Impulsivity was associated with increased heart rate to an auditory attention task in men [7]. In domestic violence offenders, trait impulsivity was associated with increased skin conductance response (SCR) during a preparatory period to confront stress [8]. In a double-blind study of individual differences in acute subjective responses to amphetamine, high trait impulsivity was significantly associated with greater arousal and euphoria [9]. Gamblers showed increased heart rate during the game, in positive correlation with subjective report of arousal and sensation seeking [10]. Frequent gamblers were found to have significantly higher levels of arousal, which continue to rise after play, in contrast to infrequent and non-gamblers [11]. In children with ADHD performing a delayed reaction time task, reward evoked higher SCR and led to more impulsive responses, as compared to control participants [12]. High impulsive men exhibited slower heart rate under rest along with greater reactivity to a behavioral challenge [7]. Also in support of the hypothesis are studies reporting increased orienting ERPs during sensory stimulation in impulsive individuals [13, 14].

On the other hand, no studies to our knowledge have examined the neural bases of arousal regulation in association with impulsivity. Here, we addressed this issue by studying 26 healthy adults in a stop signal task in combination with functional magnetic resonance imaging (fMRI) and concurrent recording of skin conductance. First, we examined whether impulsivity, as assessed by Barratt Impulsivity Scale (BIS-11), is associated with skin conductance level (SCL) and SCR to infrequent, salient events. Second, our previous fMRI study demonstrated that the time course of the ventromedial prefrontal cortex (vmPFC) is negatively correlated with SCL and the strength of Granger causality of vmPFC on SCL is negatively correlated with SCR to salient stimuli, suggesting a prefrontal cortical mechanism of arousal regulation [15]. Our second aim was to examine how inter-subject variation in impulsivity relates to vmPFC regulation of skin conductance. Third, there is evidence for gender difference in the association between trait impulsivity and risky behavior [16–20]. Thus, we examined whether the association between impulsivity and physiological arousal as well as the neural basis of arousal regulation varies between men and women.

## Materials and Methods

### Subjects, behavioral task, and Barratt Impulsivity Scale

Twenty-six adult healthy subjects (14 males, 18–52 years of age, all right-handed and using their right hand to respond) participated in this study (Table 1). This sample comprised 20 of the 24 participants reported in our previous work (Zhang et al., 2014; the other 4 were not assessed for impulsivity and thus not included) and 6 new subjects, all studied under an identical protocol. All participants denied medical including neurological illnesses, history of head injuries, current use of any medications, or use of any psychotropic medications in the past year. They were also free of any psychiatric diagnoses as assessed with the Structured Clinical Interview for Diagnostic and Statistical Manual Disorders [21], denied use of illicit substances, and showed a negative urine toxicology test on the day of fMRI. All subjects were paid to participate and signed a written consent after details of the study were explained, in accordance to a protocol approved by the Yale Human Investigation Committee.

**Table 1:** Demographics of the subjects.

Subject characteristic	All (n = 26)	Male (n = 14)	Female (n = 12)	p-value
Ages (years)	30.6 ± 10.3	31.1 ± 11.2	29.9 ± 9.5	0.78*
Race (EA/AA/Others)	21/3/2	12/1/1	9/2/1	0.74^
Education (years)	16.6 ± 3.2	16.5 ± 3.9	16.8 ± 1.9	0.77*

Note: values are mean ± S.D.; p-values are from statistic comparison between males and females

*two-tailed two-sample t test

^chi-square test

We employed a simple reaction time (RT) task in this stop-signal paradigm, as described in details previously [22–24]. Briefly, there were two trial types: “go” (~75%) and “stop” (~25%), randomly intermixed. A small dot appeared on the screen to engage attention at the beginning of a go trial. After a randomized time interval (fore-period) between 1 and 5 s, the dot turned into a circle, prompting the subjects to quickly press a button. The circle vanished at button press or after 1 s had elapsed, whichever came first, and the trial terminated. A premature button press prior to the appearance of the circle also terminated the trial. In a stop trial, an “X,” the “stop” signal, appeared and replaced the go signal, instructing the subjects to withhold button press. Likewise, a trial terminated at button press or after 1 s had elapsed. There was an inter-trial-interval of 8 s to allow adequate spacing between events of interest and identification of electrodermal responses associated with these events. The time interval between go and stop signals or stop signal delay (SSD) started at 200 ms and varied from one stop trial to the next according to a staircase procedure, increasing and decreasing by 64 ms, each after a successful and failed stop trial [25, 26]. With the staircase procedure, a “critical” SSD could be computed that represents the time delay required for the subject to succeed in half of the stop trials [25]. The stop signal reaction time was computed by subtracting the critical SSD from median go RT. Subjects were instructed to respond to the go signal quickly while keeping in mind that a stop signal could come up in a small number of trials [27]. With the staircase procedure we anticipated that the subjects would succeed in withholding their response in approximately half of the stop trials. After a practice session outside the scanner, each subject completed six 10-min runs of the task in the scanner.

Individual impulsivity was quantified from the total score on the Barratt Impulsiveness Scale, version 11 or BIS-11 [28, 29]. Three subscores of the BIS-11 were also computed for “attentional impulsivity” or an inability to focus, “motor impulsivity” or acting without prior thought, and “nonplanning impulsivity” or not thinking and planning carefully [29]. Each subscale comprises 10 questions with some items to be reverse-scored to control for response bias. The BIS-11 is widely used in personality and clinical research to capture individual tendency toward rash and risk behaviors. The BIS subscores are missing for a male subject. Thus, in the following analysis, only 25 subjects were used for subscores related analysis.

### Skin conductance acquisition and analysis

With a Biopac MP150 system, skin conductance was continuously recorded during fMRI from the palmer surfaces of the index and middle fingers of the left hand. The Biopac system used a AcqKnowledge 4.1 software (Biopac Systems, USA) and the Biopac electrodermal activity amplifier module (Galvanic Skin Response [GSR] 100c) set at a channel sampling rate of 31 Hz and a gain of 5 μSiemens (μS) per volt (resulting in a resolution of 0.0015 μS). Recording of skin conductance is synchronized with behavioral task and image acquisition. A smoothing function with a moving average of 500ms was applied in order to eliminate high-frequency noise [30]. The SCL was computed by resampling the skin conductance waveform to match the TR (2 s) used in functional imaging data acquisition and analysis [31, 32]. Because each individual trial lasted longer than 10 s, we used a 10-s window aligned with go signal onset to compute the SCR for each trial. The SCR was computed as the onset-to-peak amplitude difference in skin conductance in this 10-s window as in a previous study [33].

### Skin conductance response: impulsivity and gender differences

We computed the skin conductance responses (SCR) to go (G), stop success (SS), and stop error (SE) trials. In an analysis of variance we examined the main effects of trial type and gender and their interaction effect on the magnitude of SCR, followed by planned comparisons. With Pearson regressions we examined the correlation between each SCR and BIS-11 total score for men and women combined as well as separately. In cases where the results were statistically significant, we re-examined the correlation of SCR to the attention, motor, and nonplanning subscores of BIS-11.

### Imaging protocol and data preprocessing

Conventional T1-weighted spin echo sagittal anatomical images were acquired for slice localization using a 3T scanner (Siemens Trio). Anatomical images of the functional slice locations were next obtained with spin echo imaging in the axial plane parallel to the anterior commissure–posterior commissure (AC–PC) line with TR = 300 ms, TE = 2.5 ms, bandwidth = 300 Hz/pixel, flip angle = 60°, field of view = 220 × 220 mm, matrix = 256 × 256, 32 slices with slice thickness = 4 mm and no gap. Functional, blood oxygenation level-dependent (BOLD) signals were then acquired with a single-shot gradient echo echoplanar imaging (EPI) sequence. Thirty-two axial slices parallel to the AC–PC line covering the whole brain were acquired with TR = 2000 ms, TE = 25 ms, bandwidth = 2004 Hz/pixel, flip angle = 85°, field of view = 220 × 220 mm, matrix = 64 × 64, 32 slices with slice thickness = 4 mm and no gap.

Brain imaging data were preprocessed using Statistical Parametric Mapping version 8 (Wellcome Department of Imaging Neuroscience, University College London, U.K.). Images from the first five TRs at the beginning of each session/run were discarded to enable the signal to achieve steady-state equilibrium between radiofrequency pulsing and relaxation. Images of each individual subject were first corrected for slice timing and realigned (motion corrected). A mean functional image volume was constructed for each subject for each run from the realigned image volumes. These mean images were normalized to an MNI (Montreal Neurological Institute) EPI template with affine registration followed by nonlinear transformation [34]. The normalization parameters determined for the mean functional volume were then applied to the corresponding functional image volumes for each subject. Finally, images were smoothed with a Gaussian kernel of 8 mm at Full Width at Half Maximum.

Additional preprocessing was applied to reduce spurious BOLD variances that were unlikely to reflect neuronal activity [35, 36]. The sources of spurious variance were removed through linear regression by including the signal from the ventricular system, the white matter, and the whole brain, in addition to the six parameters obtained by rigid body head motion correction. First-order derivatives of the whole brain, ventricular and white matter signals were also included in the regression.

### Voxelwise linear correlation with skin conductance

We computed for individual subjects the correlation coefficient between the skin conductance level (SCL) and the time courses of each voxel for the whole brain. Note that the skin conductance impulse response function is very close in shape and latency to that of the canonical hemodynamic response function [31, 37]. Thus, SCL could be cross correlated with BOLD signals without additional processing. We then converted these individual correlation maps, which were not normally distributed, to z score maps by Fisher’s z transform: *z* = 0.5log_*e*_[(1+*r*)/(1−*r*)]. The z maps were used in the second level random effects analysis [38]. A one-sample t-test was applied to the ‘z maps’ across all subjects to identify regional activities correlated to skin conductance.

Functional regions of interest (ROIs) were defined based on activated clusters at a corrected threshold from whole-brain analysis. All voxel activations were presented in MNI coordinates.

### Granger causality analysis of brain activity and SCL time course

BOLD and skin conductance signals were examined with Granger causality analysis (GCA, [39]), which has been widely used to describe “causal” influence between sets of EEG or fMRI time series [40–46], as described in details in our previous studies [47–49]. Briefly, we used multivariate autoregressive (MAR) modeling [46, 50] to perform GCA. In an unrestricted model of the time series

$$Y(t)=\sum_{i=1}^{p}A_{i}Y(t-i)+\varepsilon (t),t=1,2,\ldots ,T,$$ (1)

*Y*(*t*) is a column vector [*y*_1_(*t*), *y*_2_(*t*),…, *y*_*n*_(*t*)] in which each element *y*_*j*_(*t*), *j* = 1,2,…,*n*, is the average time series of a region of interest (ROI) or SCL at time point *t*; *T* is the number of time points; *n* is the number of ROI/SCL; and *ε*(*t*) is a column vector [*ε*_1_(*t*), *ε*_2_(*t*),…, *ε*_*n*_(*t*)] of residuals at time point *t*. The model order is represented by *p* and *A*_*i*_ is a *n*-by-*n* matrix given by

$$A_{i}=\left[\begin{array}{cccc} a_{11}^{\left(i\right)} & a_{12}^{\left(i\right)} & \cdots & a_{1n}^{\left(i\right)}\\ a_{21}^{\left(i\right)} & a_{22}^{\left(i\right)} & \cdots & a_{2n}^{\left(i\right)}\\ \vdots & \vdots & \ddots & \vdots \\ a_{n1}^{\left(i\right)} & a_{n2}^{\left(i\right)} & \cdots & a_{nn}^{\left(i\right)} \end{array}\right],i=1,2,\ldots ,p,$$ (2)

estimated by ordinary least squares [51]. To determine the model order, we employed the Bayesian Information Criterion [52, 53]. The application of MAR modeling required that each ROI or SCL was covariance stationary, which we examined with the Augmented Dickey Fuller (ADF) test [54]. The ADF test verified that there was no unit root in the modeled time series. To test whether variable *x* Granger causes *y*, where *x*, *y* ∈ *Y*(*t*), *x* ≠ *y*, we computed the regression Eq (1) without variable *x* (the restricted model) and obtained the residual sum of squares *RSS*_*r*_ of variable *y*. The residual sum of squares of *y* is given by $RSS=\sum_{t=1}^{T}{\left(y(t)-\hat{y}(t)\right)}^{2}=\sum_{t=1}^{T}\varepsilon {\left(t\right)}^{2}$, where $\hat{y}$ represents the predicted value of *y*. These residuals were used to compute the Granger causality strength measures (*F-*values) of each possible connection between ROIs and skin conductance [54]:

F=(RSSr−RSSur)pRSSur(T−2p−1), (3)

where *RSS*_*ur*_ is the residual sum of squares of variable *y* in the unrestricted model. We tested the significance of the Granger causality between time series by an *F* test and used binomial test to assess statistical significance in group analysis as described in details earlier [47–49]. For each connection, we counted the number of subjects that had significant connections and estimated its significance using a binomial distribution with parameters n = 26, and p = q = 0.5 (same probability to observe a connection or not). For each subject, we had a total of 1770 (295 x 6) time points for Granger causality analysis.

To assess how the strength of Granger causality relates to event-evoked arousal, we examined the correlation across subjects between the causality strength measures (*F*-values) and stimulus-evoked SCR with a linear regression.

### Impulsivity and ventromedial prefrontal cortical regulation of skin conductance

As described above, our earlier work with GCA suggested a regulatory role of the ventromedial prefrontal cortex (vmPFC) on skin conductance [15]. The strength of Granger causality was inversely correlated with the SCR to stop trials. Thus, here, we examined whether this Granger causality is correlated to BIS-11 total score in men and women combined and separately.

## Results

### Impulsivity, behavioral performance and skin conductance response

Across subjects, BIS-11 total scores ranged from 42 to 85 with a mean ± S.D. of 60.2 ± 11.9. In pairwise correlation, the subscale scores showed a correlation between attention and motor impulsivity (p = 0.00007), between attention and non-planning impulsivity (p = 0.003), but not between motor and non-planning impulsivity (p = 0.06). Men and women did not differ in either total (men: 63 ± 12; women 56 ± 12) or subscale scores (all p’s > 0.14).

Across subjects, participants responded in 97.8% ± 3.5% of go trials and 55.0% ± 2.9% of stop trials. The average response time of stop error trials (606 ± 92 ms) was shorter than the go response trials (669 ± 67 ms, p<0.00001, paired sample t test). The stop signal reaction time (SSRT) was 236 ± 32 ms across subjects. These results are typical of stop signal task performance. Men and women did not show a difference in SSRT (p = 0.29) nor was there a correlation between BIS-11 total score and SSRT in men and women combined (p = 0.62) or in man (p = 0.20) or women (p = 0.29) alone [55].

**Fig 1:**
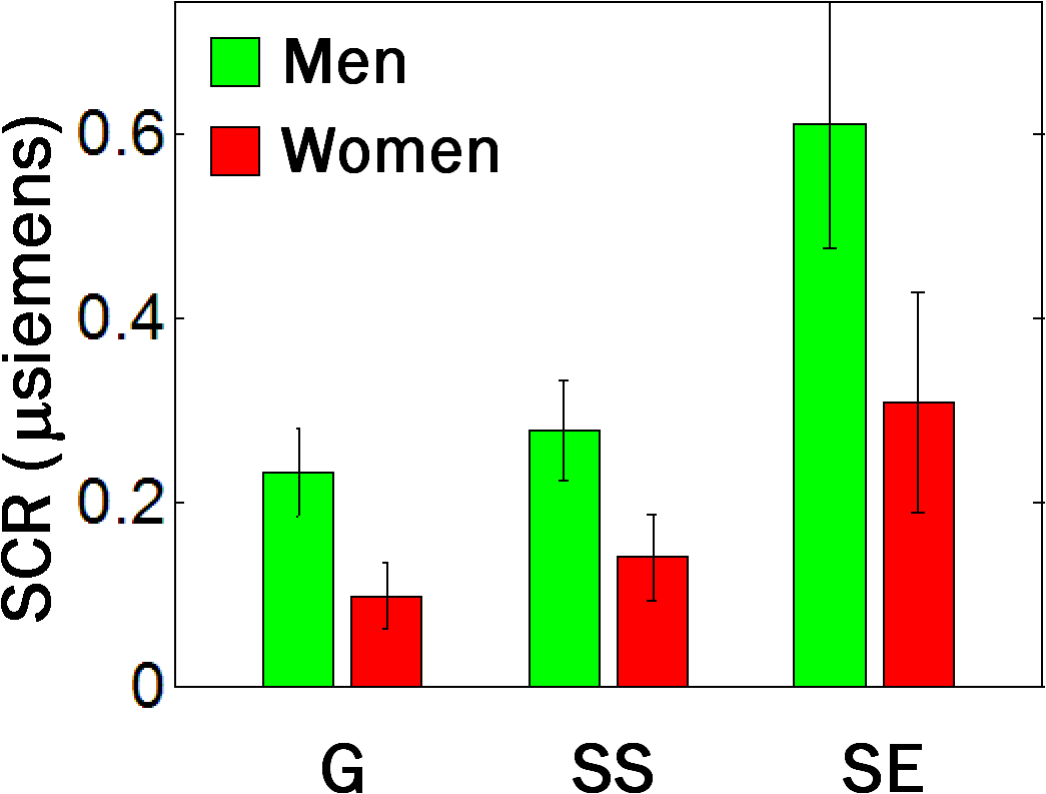
Skin conductance responses in the SST. Skin conductance responses (SCR) to go (G), stop success (SS) and stop error (SE) trials in the SST. Data bars show SCR (mean ± S.E.) for men (n = 14) and women (n = 12) separately. Across all 26 subjects, G, SS and SE trials showed significant differences in SCR (p = 0.002, trial main effect, two-way ANOVA), as did planned comparisons: G vs. SS (p = 0.02), G vs. SE (p = 0.0002), and SS vs. SE (p = 0.0002), with two-sample t tests. There was also a gender main effect with men showing greater SCR than women (p = 0.009) but no interaction effect between trial and gender (p = 0.54).

Skin conductance responses (SCR) during the SST are shown in Fig 1. As described in the Methods, we quantified the SCR by subtracting the amplitude at the baseline from the amplitude at the peak in a 10-second window after stimulus onset for each trial. Across all 26 subjects, go (G), stop success (SS) and stop error (SE) trials showed significant differences in SCR (p = 0.002, trial main effect, two-way ANOVA), as did planned comparisons: G vs. SS (p = 0.02), G vs. SE (p = 0.0002), and SS vs. SE (p = 0.0002), with two-sample t tests. There was also a gender main effect with men showing greater SCR than women (p = 0.009) but no interaction effect between trial and gender (p = 0.54).

### Impulsivity and skin conductance response

**Fig 2:**
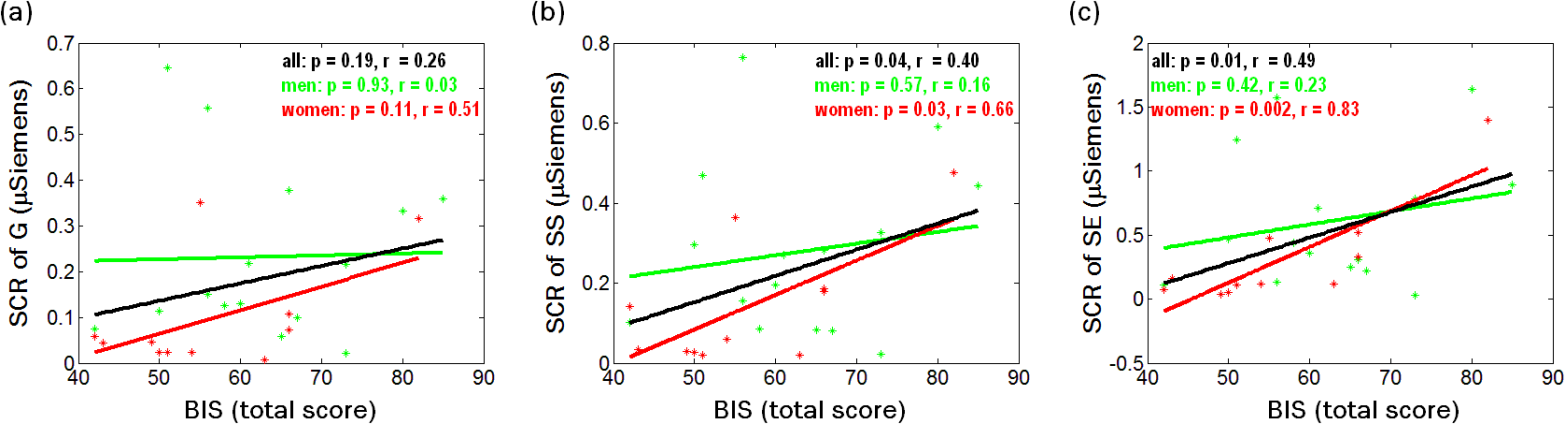
Correlation between BIS-11 total score and SCR. Correlation between BIS-11 total score and SCR to (a) G, (b) SS, and (c) SE trials across all 26 subjects (black) as well as across 14 men (green) and 12 women (red) separately.

Across subjects, BIS-11 total score correlated positively with SCR to SS and SE but not G trials (Fig 2). In regressions conducted separately for men and women, only women demonstrated significant correlations between BIS-11 and SCR to SS and SE trials (Fig 2).

We further examined the correlation between BIS-11 subscores and SCR. The results showed that SCR is related to attentional and motor but not nonplanning impulsivity in women and SCR is not significantly correlated to any of the subscores in men (Table 2).

**Table 2:** A summary of correlations between BIS-11 subscores (Attention, Motor, and Nonplanning Impulsiveness) and SCR.

	Attention		Motor		Nonplanning	
	p	r	p	r	p	r
*All subjects*:						
SCR of G	0.06	0.38	0.08	0.35	0.86	0.04
SCR of SS	**0.006**	0.54	**0.01**	0.51	0.27	0.23
SCR of SE	**0.01**	0.49	**0.002**	0.59	0.30	0.21
*Men*:						
SCR of G	0.34	0.28	0.65	0.13	0.62	0.14
SCR of SS	0.13	0.42	0.28	0.31	0.24	0.34
SCR of SE	0.25	0.33	0.18	0.38	0.27	0.31
*Women*:						
SCR of G	0.26	0.37	**0.02**	0.67	0.87	-0.06
SCR of SS	**0.04**	0.63	**0.004**	0.79	0.61	0.17
SCR of SE	**0.02**	0.68	**0.0002**	0.89	0.64	0.16

* P values are not corrected.

### Arousal related brain activation and Granger causality analysis (GCA)

**Fig 3:**
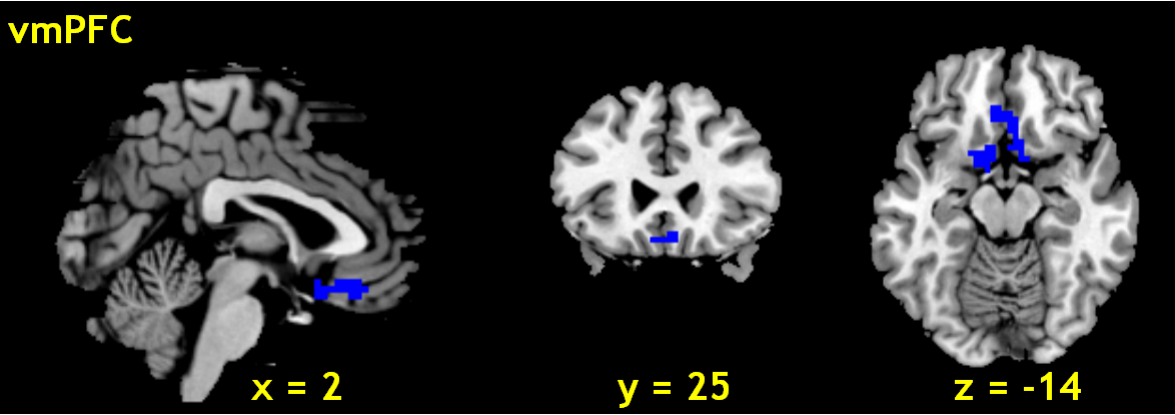
Brain region negatively correlated with skin conductance. The ventromedial prefrontal cortex (vmPFC, x = 2, y = 25, z = -14) showed significant negative correlation with skin conductance across 26 subjects (peak voxel p < 0.001 and cluster level p < 0.05, FWE corrected).

As expected, we observed that the ventromedial prefrontal cortex (vmPFC, x = 2, y = 25, z = -14) showed significant (peak voxel p < 0.001 and cluster level p < 0.05, FWE corrected) negative correlation with skin conductance (Fig 3).

The results of GCA showed that BOLD signals of the vmPFC Granger caused the SCL (p<0.05 for individual GCA and p = 0.04, binomial test for group analysis), but the SCL did not Granger cause vmPFC activity (p = 0.16). Moreover, individuals varied in the strength of Granger causality as indexed by the F value (mean ± SD: 5.0 ± 4.5; range: 0.4 to 18.6). Across all participants, Pearson regressions showed that higher Granger causality strength (*F*-values) of the vmPFC was associated with less SCR elicited to go (p = 0.008, r = -0.52), stop success (p = 0.006, r = -0.54), and stop error (p = 0.02, r = -0.47) trials during the stop signal task.

**Fig 4:**
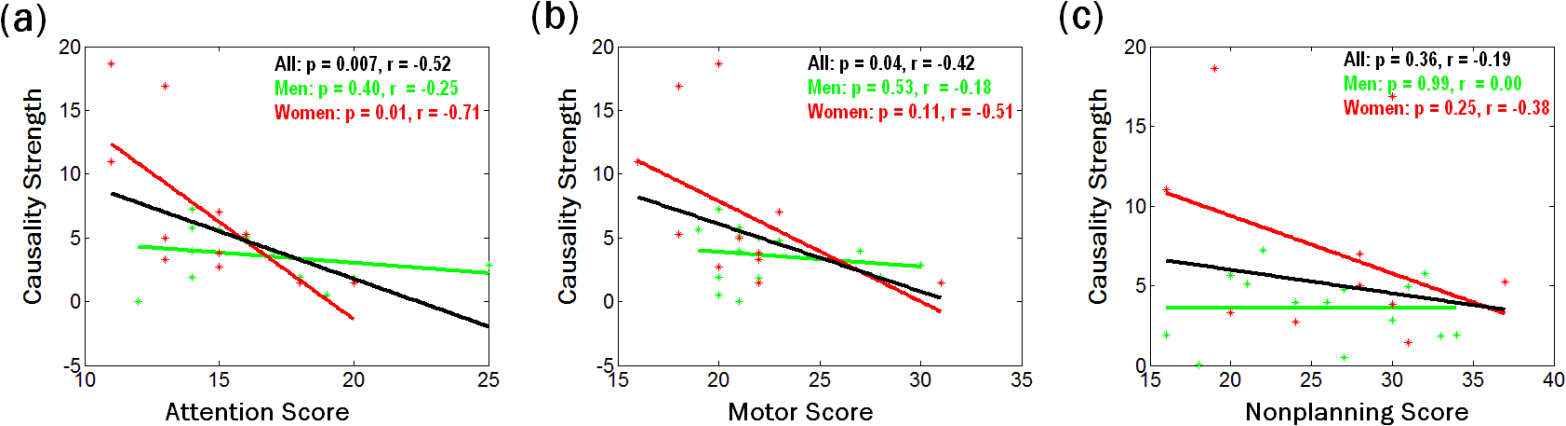
Correlation between Granger causality strength and BIS-11 sub-scores. Correlation between Granger causality strength and BIS-11 sub-scores of (a) attention, (b) motor, and (c) nonplanning across all 26 subjects (black) as well as across 14 men (green) and 12 women (red) separately.

We assessed how the strength of Granger causality of vmPFC on SCL is related to the BIS-11 total score and subscores. The results showed a significant negative correlation between the F value and total score (p = 0.04, r = -0.41) in men and women combined, but not in women (p = 0.12) or men (p = 0.54) alone. For the entire sample, F value was also significantly correlated to attention subscore (p = 0.007, r = -0.52), motor subscore (p = 0.04, r = -042) but not nonplanning subscore (p = 0.36). Analyzed separately for men and women, inattention impulsivity showed negative correlation with the Granger causality strength in women (p = 0.01, r = -0.71) but not men (p = 0.40) (Fig 4). Motor and non-planning impulsivity was not correlated with the causality strength in women or men alone.

## Discussion

The current results showed that Barratt impulsivity is associated with heightened skin conductance response (SCR) to salient events. Furthermore, Barratt impulsivity is associated with decreased ventromedial prefrontal cortical regulation of physiological arousal in women. These findings support the hypothesis of altered arousal regulation in impulsive individuals and a prefrontal mechanism underlying this association in women. These findings are of clinical importance in that impulsivity and arousal dysregulation are both biological hallmarks of addiction [56–60]. Furthermore, impulsivity is known to be a dimensional trait that varies continuously in the general population [61–63]. Unraveling the neural processes of arousal regulation would advance our understanding of the biological bases of impulsivity in health and illness. We discuss these findings in the below.

### Impulsivity and physiological arousal

As described in the above, our findings showed that impulsivity is associated with increased physiological arousal to salient events. On the other hand, a smaller number of studies reported contrasting findings. In an auditory task where participants were to detect an “odd-ball” among standard tones, individuals high on impulsivity and sensation seeking showed less SCR to the deviation [64]. In studies of acoustic startle reflex, high sensation seeking individuals demonstrate reduced startle reactivity [65, 66]. In a gambling task, electrodermal reactivity increased to losses as compared to wins and this difference is negatively correlated with trait impulsivity in adolescents [67]. Together, these studies suggest lower arousal and/or arousability in association with impulsivity. Studies of physiological arousal at rest, as indexed by skin conductance level (SCL), are fewer in number but similarly depict a conflicting picture. In children, proactive aggression and high impulsivity is associated with increased resting skin conductance [68]. However, in boys with attention deficit hyperactivity disorder, symptomatic severity is associated with lower sympathetic and higher parasympathetic activity [69].

It is not clear what may account for these discrepant findings. One potential factor is that many of these studies comprised various clinical populations or individuals who demonstrated varying extent of risky behaviors including habitual gambling and multiple sexual partners. Further, many studies focused only on men and few have considered gender in data analyses. More studies clearly are needed to address this issue.

### Impulsivity and neural bases of physiological arousal

A growing body of research employs brain imaging to unravel the neural bases of impulsivity. For instance, in otherwise healthy individuals, impulsivity is associated with increased responses of the anterior cingulate cortex and amygdala [70] and decreased responses of the anterior pre-supplementary motor area [71] to anticipation of reward, and decreased activation of the right insula and middle frontal cortex in processing salient stimuli [55]. Impulsivity, as indexed by temporal discounting, is associated with increased striatal subcortical connectivity [72]. In adolescents, impulsivity is associated with lower activity in the substantia nigra and subthalamic nucleus but higher activity in the presupplementary motor area and precentral gyrus during successful response inhibition [73]. Together, these recent findings suggest a diverse picture of cerebral functioning in link with impulsivity and multifaceted neural bases of impulsive behavior.

Changes in physiological arousal accompany attention, decision-making, affective regulation, and other motivated behaviors, processes known to be altered in impulsive individuals [74–79]. The ventromedial prefrontal cortex (vmPFC) is consistently implicated in cerebral responses to physiological arousal [37, 80–85]. It has been postulated that the vmPFC may play a role in regulating physiological arousal [32, 76, 83]. Indeed, here and earlier we demonstrated that vmPFC activity not only negatively correlates with but Granger causes skin conductance level and the strength of causality is negatively associated with SCR to salient events [15].

Furthermore, we showed that, in women, attention impulsivity is associated with decreased Granger causality in vmPFC regulation of skin conductance. This finding suggests that impulsivity-linked increases in arousal responses to saliency may be related to deficient prefrontal control in women. The stronger the regulatory influence of the vmPFC, the less the skin conductance responses to infrequent events, and this neural mechanism of control is disrupted in impulsive women.

### Gender differences in arousal regulation and the role of impulsivity

Men demonstrated significantly higher SCR to event onsets in the stop signal task, as compared to women. This finding is consistent with a number of studies of gender differences in arousal responses. For instance, in contrast to women, men are more susceptible to framing effects and exhibited greater SCR concurrent with defensive or orienting responses in an Ultimatum game [86]. An imaging study examined stress circuit activities while participants viewed negative valence/high arousal versus neutral stimuli [87]. Men showed greater signal changes than women in late follicular/luteal phases but not women during follicular phase, suggesting that stress-elicited gender differences in arousal response are mediated by sexual hormones. During a Stroop test, men showed higher systolic blood pressure and epinephrine plasma concentrations than women [88]. When confronted with angry or fearful faces, men showed greater anterior cingulate and visual cortical responses in association with heightened vigilance than women [89]. Compared to black women, black men showed increased sympathetic responses including elevated epinephrine level during recall of anger and negative affect [90]. During viewing of affective pictures, the relationships between self-rated arousal, blood pressure and cardiac stroke volume were mainly exhibited by men, suggesting that increases in the sympathetic inotropic effect to the heart with self-rated arousal may be larger in men than in women [91].

Men and women did not differ in BIS-11 total score or any of the subscores, and inter-subject variation in Barratt impulsivity is related to the SCR in women but not men. These results suggest a few non-exclusive possibilities. First, as we discussed in an earlier work, BIS-11 addresses only certain dimensions and may not capture the full spectrum of impulsivity trait [55]. Future work should include other impulsivity measures such as Eysenck's scales, which considers impulsiveness and venturesomeness [4] and UPPS (urgency, premeditation, perseverance, and sensation seeking) impulsive behavior scale [92]. Second, impulsivity may not be the most important personality trait in determining arousal responses in men. Previous studies have suggested reward sensitivity may play a critical role in behavioral activation in men. For instance, men showed greater sensitivity to reward as assessed by the Sensitivity to Punishment and Sensitivity to Reward Questionnaire [93–102]. Reward sensitivity and anxiety each predicts risky driving in men and women [103–105]. D-Amphetamine increased risk behavior in men with high reward sensitivity, but did not affect risk-taking in women [106]. Novelty seeking with nicotine reinforcement and reward is directly related in men but inversely or unrelated in women [107]. Testosterone mediates neural responses to reward related processing in young children, which may influence behavioral approach tendencies later in life [108]. Imaging studies including those of gender differences in structural and functional cerebral bases of reward processing have also broadly suggested a role of reward sensitivity in rash actions [62, 109–111]. More studies are warranted to address the influence of these other personality traits on arousal regulation and impulsive behavior.

### Limitations of the study and conclusions

There are a few limitations to consider in the current study. First, the sample size of the current study is small and we did not consider correcting for multiple comparisons in statistical tests. The results, particularly those of gender differences, should be considered preliminary. Second, as described above, BIS-11 may not capture all of the critical dimensions of impulsivity. Thus, for instance, the finding of a lack of correlation between BIS score and SSRT, while consistent with our earlier study of a much larger sample size showing only a trend-level correlation [55] and other work [112], does not negative the relationship between impulsivity and inhibitory control. A recent study showed that UPPS subdomain urgency but not BIS best explained inter-individual variability in SSRT [113]. It is important to address how other dimensions of impulsivity may be related to the neural processes of arousal regulation. Third, multiple personality features may interact to determine arousal in response to saliency and risky behavior. Future studies should include assessment of anxiety and reward sensitivity and examine gender- shared and specific mediators of psychological and neural processes that dispose individuals to risky behavior. Fourth, while skin conductance change is linked to impulsivity, the heightened arousal response likely represents a physiological analogue and the neural processes that “drive” impulsive behavior remains to be determined.

To conclude, we reported a positive association between Barratt impulsivity and increased skin conductance response (SCR) to stop trials in the stop signal task. The increase in SCR to salient events is correlated with diminished ventromedial prefrontal cortical regulation of skin conductance in women (but not in men) higher in impulsivity. These results may advance our understanding of gender-based neural processes of risky behavior.

## Supporting Information

S1 File Original data and image/ROI format for brain region of vmPFC. (ZIP)

## References

[1] Humphreys MS, Revelle W. Personality, motivation, and performance: a theory of the relationship between individual differences and information processing. Psychological review. 1984;91(2):153–84. . 6571423

[2] Barratt ES. Impulsiveness subtraits: arousal and information processing In: Spence CE, Izard CE, editors. Motivation, emotion, and personality. New York: Elsevier Science Publishers; 1985 p. 137–46.

[3] Eysenck HJ. The biological basis of personality Springfield: Thomas 1967.

[4] Eysenck HJ, Eysenck MW. Personality and individual differences New York: Plenum 1985.

[5] Zuckerman M. Psychobiology of personality Cambridge: Cambridge University Press 1991.

[6] Derefinko KJ, Peters JR, Eisenlohr-Moul TA, Walsh EC, Adams ZW, Lynam DR. Relations between trait impulsivity, behavioral impulsivity, physiological arousal, and risky sexual behavior among young men. Archives of sexual behavior. 2014;43(6):1149–58. 10.1007/s10508-014-0327-x 24958252 PMC4134401

[7] Mathias CW, Stanford MS. Impulsiveness and arousal: heart rate under conditions of rest and challenge in healthy males. Personality and Individual Differences. 2003;35(2):355–71.

[8] Romero-Martinez A, Lila M, Williams RK, Gonzalez-Bono E, Moya-Albiol L. Skin conductance rises in preparation and recovery to psychosocial stress and its relationship with impulsivity and testosterone in intimate partner violence perpetrators. Int J Psychophysiol. 2013;90(3):329–33. 10.1016/j.ijpsycho.2013.10.003 . 24140253

[9] Kirkpatrick MG, Johanson CE, de Wit H. Personality and the acute subjective effects of d-amphetamine in humans. Journal of psychopharmacology. 2013;27(3):256–64. 10.1177/0269881112472564 23343596 PMC4241296

[10] Coventry KR, Hudson J. Gender differences, physiological arousal and the role of winning in fruit machine gamblers. Addiction. 2001;96(6):871–9. 10.1080/09652140020050997 . 11399218

[11] Moodie C, Finnigan F. A comparison of the autonomic arousal of frequent, infrequent and non-gamblers while playing fruit machines. Addiction. 2005;100(1):51–9. 10.1111/j.1360-0443.2005.00942.x . 15598192

[12] Firestone P, Douglas V. The effects of reward and punishment on reaction times and autonomic activity in hyperactive and normal children. Journal of abnormal child psychology. 1975;3(3):201–16. . 1214031 10.1007/BF00916751

[13] Carrillo-de-la-Pena MT, Barratt ES. Impulsivity and ERP augmenting/reducing. Personality and Individual Differences. 1993;15(1):25–32.

[14] Houston RJ, Stanford MS. Mid-latency evoked potentials in self-reported impulsive aggression. Int J Psychophysiol. 2001;40(1):1–15. . 11166104 10.1016/s0167-8760(00)00120-3

[15] Zhang S, Hu S, Chao HH, Ide JS, Luo X, Farr OM, et al Ventromedial prefrontal cortex and the regulation of physiological arousal. Soc Cogn Affect Neurosci. 2014;9(7):900–8. 10.1093/scan/nst064 23620600 PMC4090954

[16] DeVito EE, Meda SA, Jiantonio R, Potenza MN, Krystal JH, Pearlson GD. Neural correlates of impulsivity in healthy males and females with family histories of alcoholism. Neuropsychopharmacology: official publication of the American College of Neuropsychopharmacology. 2013;38(10):1854–63. 10.1038/npp.2013.92 23584260 PMC3746701

[17] Diekhof EK, Keil M, Obst KU, Henseler I, Dechent P, Falkai P, et al A functional neuroimaging study assessing gender differences in the neural mechanisms underlying the ability to resist impulsive desires. Brain research. 2012;1473:63–77. 10.1016/j.brainres.2012.07.010 . 22814146

[18] Li CS, Zhang S, Duann JR, Yan P, Sinha R, Mazure CM. Gender Differences in Cognitive Control: an Extended Investigation of the Stop Signal Task. Brain Imaging Behav. 2009;3(3):262–76. 10.1007/s11682-009-9068-1 19701485 PMC2728908

[19] Perry RI, Krmpotich T, Thompson LL, Mikulich-Gilbertson SK, Banich MT, Tanabe J. Sex modulates approach systems and impulsivity in substance dependence. Drug and alcohol dependence. 2013;133(1):222–7. 10.1016/j.drugalcdep.2013.04.032 23725607 PMC3786050

[20] Winhusen T, Lewis D. Sex differences in disinhibition and its relationship to physical abuse in a sample of stimulant-dependent patients. Drug and alcohol dependence. 2013;129(1–2):158–62. 10.1016/j.drugalcdep.2012.09.014 23062872 PMC3563925

[21] First MB, Spitzer RL, Williams JBW, Gibbon M. Structured Clinical Interview for DSM-IV (SCID). Washington, DC: American Psychiatric Association 1995.

[22] Chao HH, Luo X, Chang JL, Li CS. Activation of the pre-supplementary motor area but not inferior prefrontal cortex in association with short stop signal reaction time—an intra-subject analysis. BMC Neurosci. 2009;10:75 10.1186/1471-2202-10-75 19602259 PMC2719646

[23] Li CS, Chao HH, Lee TW. Neural correlates of speeded as compared with delayed responses in a stop signal task: An indirect analog of risk taking and association with an anxiety trait. Cereb Cortex. 2009;19(4):839–48. 10.1093/cercor/bhn132 18678764 PMC2722793

[24] Zhang S, Li CS. Functional networks for cognitive control in a stop signal task: Independent component analysis. Hum Brain Mapp. 2012a;33(1):89–104. 10.1002/hbm.21197 21365716 PMC3674850

[25] Levitt H. Transformed up-down methods in psychoacoustics. J Acoust Soc Am. 1971;49(2):Suppl 2:467+. . 5541744

[26] De Jong R, Coles MG, Logan GD, Gratton G. In search of the point of no return: the control of response processes. J Exp Psychol Hum Percept Perform. 1990;16(1):164–82. . 2137517 10.1037/0096-1523.16.1.164

[27] Li CS, Huang C, Yan P, Paliwal P, Constable RT, Sinha R. Neural Correlates of Posterror Slowing during a Stop Signal Task: A Functional Magnetic Resonance Imaging Study. J Cogn Neurosci. 2008;20(6):1021–9. 10.1162/jocn.2008.20071 18211230 PMC2597347

[28] Barratt ES, Patton JH. Impulsivity: cognitive behavioral, and psychophysiological correlates. In: Zuckerman M, editor. Biological Bases of Impulsiveness and Sensation Seeking. Erlbaum, Hillsdale, NJ1983.

[29] Patton JH, Stanford MS, Barratt ES. Factor structure of the Barratt impulsiveness scale. Journal of clinical psychology. 1995;51(6):768–74. . 8778124 10.1002/1097-4679(199511)51:6<768::aid-jclp2270510607>3.0.co;2-1

[30] Figner B, Murphy RO. Using skin conductance in judgment and decision making research In: Schulte-Mecklenbeck M, Kuehberger A, Ranyard R, editors. A handbook of process tracing methods for decision research. New York, NY: Psychology Press; 2010 p. 163–84.

[31] Patterson JC 2nd, Ungerleider LG, Bandettini PA. Task-independent functional brain activity correlation with skin conductance changes: an fMRI study. Neuroimage. 2002;17(4):1797–806. . 12498753 10.1006/nimg.2002.1306

[32] Critchley HD, Elliott R, Mathias CJ, Dolan RJ. Neural activity relating to generation and representation of galvanic skin conductance responses: a functional magnetic resonance imaging study. J Neurosci. 2000;20(8):3033–40. . 10751455 10.1523/JNEUROSCI.20-08-03033.2000 PMC6772223

[33] Zhang S, Hu S, Chao HH, Luo X, Farr OM, Li CS. Cerebral correlates of skin conductance responses in a cognitive task. Neuroimage. 2012;62(3):1489–98. 10.1016/j.neuroimage.2012.05.036 22634217 PMC3408848

[34] Ashburner J, Friston KJ. Nonlinear spatial normalization using basis functions. Hum Brain Mapp. 1999;7(4):254–66. . 10408769 10.1002/(SICI)1097-0193(1999)7:4<254::AID-HBM4>3.0.CO;2-G PMC6873340

[35] Zhang S, Li CS. Functional connectivity mapping of the human precuneus by resting state fMRI. Neuroimage. 2012b;59(4):3548–62. 10.1016/j.neuroimage.2011.11.023 22116037 PMC3288461

[36] Fox MD, Snyder AZ, Vincent JL, Corbetta M, Van Essen DC, Raichle ME. The human brain is intrinsically organized into dynamic, anticorrelated functional networks. Proc Natl Acad Sci U S A. 2005;102(27):9673–8. . 15976020 10.1073/pnas.0504136102 PMC1157105

[37] Nagai Y, Critchley HD, Featherstone E, Trimble MR, Dolan RJ. Activity in ventromedial prefrontal cortex covaries with sympathetic skin conductance level: a physiological account of a "default mode" of brain function. Neuroimage. 2004;22(1):243–51. . 15110014 10.1016/j.neuroimage.2004.01.019

[38] Penny WD, Holmes AP, Friston K. Random-effects analysis In: Frackowiak R, Frith C, Dolan RJ, Friston K, Price C, Zeki S, et al, editors. Human Brain Function: Academic Press; 2004 p. 843–50.

[39] Granger CWJ. Investigating causal relations by econometric models and cross-spectral methods. Econometrica. 1969;37:424.

[40] Ding M, Bressler SL, Yang W, Liang H. Short-window spectral analysis of cortical event-related potentials by adaptive multivariate autoregressive modeling: data preprocessing, model validation, and variability assessment. Biol Cybern. 2000;83(1):35–45. . 10933236 10.1007/s004229900137

[41] Wen X, Yao L, Liu Y, Ding M. Causal interactions in attention networks predict behavioral performance. J Neurosci. 2012;32(4):1284–92. 10.1523/JNEUROSCI.2817-11.2012 22279213 PMC6796284

[42] Roebroeck A, Formisano E, Goebel R. Mapping directed influence over the brain using Granger causality and fMRI. Neuroimage. 2005;25(1):230–42. . 15734358 10.1016/j.neuroimage.2004.11.017

[43] Abler B, Roebroeck A, Goebel R, Hose A, Schonfeldt-Lecuona C, Hole G, et al Investigating directed influences between activated brain areas in a motor-response task using fMRI. Magn Reson Imaging. 2006;24(2):181–5. . 16455407 10.1016/j.mri.2005.10.022

[44] Deshpande G, Hu X, Stilla R, Sathian K. Effective connectivity during haptic perception: a study using Granger causality analysis of functional magnetic resonance imaging data. Neuroimage. 2008;40(4):1807–14. 10.1016/j.neuroimage.2008.01.044 18329290 PMC2483676

[45] Stilla R, Deshpande G, LaConte S, Hu X, Sathian K. Posteromedial parietal cortical activity and inputs predict tactile spatial acuity. J Neurosci. 2007;27(41):11091–102. . 17928451 10.1523/JNEUROSCI.1808-07.2007 PMC6672842

[46] Sato JR, Takahashi DY, Arcuri SM, Sameshima K, Morettin PA, Baccala LA. Frequency domain connectivity identification: an application of partial directed coherence in fMRI. Hum Brain Mapp. 2009;30(2):452–61. . 18064582 10.1002/hbm.20513 PMC6871135

[47] Duann JR, Ide JS, Luo X, Li CS. Functional connectivity delineates distinct roles of the inferior frontal cortex and presupplementary motor area in stop signal inhibition. J Neurosci. 2009;29(32):10171–9. 10.1523/JNEUROSCI.1300-09.2009 19675251 PMC2769086

[48] Ide JS, Li CS. A cerebellar thalamic cortical circuit for error-related cognitive control. Neuroimage. 2011a;54(1):455–64. 10.1016/j.neuroimage.2010.07.042 20656038 PMC2962720

[49] Ide JS, Li CS. Error-related functional connectivity of the habenula in humans. Front Hum Neurosci. 2011b;5:25 10.3389/fnhum.2011.00025 21441989 PMC3060701

[50] Harrison L, Penny WD, Friston K. Multivariate autoregressive modeling of fMRI time series. Neuroimage. 2003;19(4):1477–91. . 12948704 10.1016/s1053-8119(03)00160-5

[51] Seth AK. A MATLAB toolbox for Granger causal connectivity analysis. J Neurosci Methods. 2010;186(2):262–73. 10.1016/j.jneumeth.2009.11.020 19961876

[52] Gentle JE, Härdle W, Mori Y. Handbook of computational statistics: concepts and methods: Springer; 2004.

[53] Schwarz G. Estimating the dimension of a model. Annals of Statistics. 1978;6(2):461–4.

[54] Hamilton JD. Time Series Analysis. Princeton, NJ: Princeton University Press; 1994.

[55] Farr OM, Hu S, Zhang S, Li CS. Decreased saliency processing as a neural measure of Barratt impulsivity in healthy adults. Neuroimage. 2012;63(3):1070–7. 10.1016/j.neuroimage.2012.07.049 22885245 PMC3472158

[56] Acton GS. Measurement of impulsivity in a hierarchical model of personality traits: implications for substance use. Substance use & misuse. 2003;38(1):67–83. . 12602807 10.1081/ja-120016566

[57] Berridge CW, Arnsten AF. Psychostimulants and motivated behavior: Arousal and cognition. Neuroscience and biobehavioral reviews. 2013. 10.1016/j.neubiorev.2012.11.005 . 23164814

[58] Boutrel B, de Lecea L. Addiction and arousal: the hypocretin connection. Physiology & behavior. 2008;93(4–5):947–51. 10.1016/j.physbeh.2007.11.022 18262574 PMC4307930

[59] Coskunpinar A, Cyders MA. Impulsivity and substance-related attentional bias: a meta-analytic review. Drug and alcohol dependence. 2013;133(1):1–14. 10.1016/j.drugalcdep.2013.05.008 . 23746428

[60] Paulus MP, Stewart JL. Interoception and drug addiction. Neuropharmacology. 2014;76 Pt B:342–50. 10.1016/j.neuropharm.2013.07.002 23855999 PMC3858461

[61] Eysenck SB, Eysenck HJ. The place of impulsiveness in a dimensional system of personality description. The British journal of social and clinical psychology. 1977;16(1):57–68. . 843784 10.1111/j.2044-8260.1977.tb01003.x

[62] Hahn T, Dresler T, Ehlis AC, Plichta MM, Heinzel S, Polak T, et al Neural response to reward anticipation is modulated by Gray's impulsivity. Neuroimage. 2009;46(4):1148–53. 10.1016/j.neuroimage.2009.03.038 . 19328237

[63] Muller VI, Langner R, Cieslik EC, Rottschy C, Eickhoff SB. Interindividual differences in cognitive flexibility: influence of gray matter volume, functional connectivity and trait impulsivity. Brain structure & function. 2014. 10.1007/s00429-014-0797-6 . 24878823 PMC4981636

[64] De Pascalis V, Valerio E, Santoro M, Cacace I. Neuroticism-Anxiety, Impulsive-Sensation Seeking and autonomic responses to somatosensory stimuli. Int J Psychophysiol. 2007;63(1):16–24. . 16899317 10.1016/j.ijpsycho.2006.06.004

[65] Giakoumaki SG, Roussos P, Tsapakis EM, Koiliari E, Pasparakis E, Zouraraki C, et al Cognitive and personality analysis of startle reactivity in a large cohort of healthy males. Biol Psychol. 2013;94(3):582–91. 10.1016/j.biopsycho.2013.09.005 . 24051230

[66] Roussos P, Giakoumaki SG, Bitsios P. Cognitive and emotional processing in high novelty seeking associated with the L-DRD4 genotype. Neuropsychologia. 2009;47(7):1654–9. 10.1016/j.neuropsychologia.2009.02.005 . 19397860

[67] Stankovic A, Fairchild G, Aitken MR, Clark L. Effects of psychosocial stress on psychophysiological activity during risky decision-making in male adolescents. Int J Psychophysiol. 2014;93(1):22–9. 10.1016/j.ijpsycho.2013.11.001 . 24252595

[68] Scarpa A, Haden SC, Tanaka A. Being hot-tempered: autonomic, emotional, and behavioral distinctions between childhood reactive and proactive aggression. Biol Psychol. 2010;84(3):488–96. 10.1016/j.biopsycho.2009.11.006 . 19941933

[69] Wang TS, Huang WL, Kuo TB, Lee GS, Yang CC. Inattentive and hyperactive preschool-age boys have lower sympathetic and higher parasympathetic activity. The journal of physiological sciences: JPS. 2013;63(2):87–94. 10.1007/s12576-012-0238-3 . 23076674 PMC10717439

[70] Kerr KL, Avery JA, Barcalow JC, Moseman SE, Bodurka J, Bellgowan PS, et al. Trait impulsivity is related to ventral ACC and amygdala activity during primary reward anticipation. Soc Cogn Affect Neurosci. 2014. 10.1093/scan/nsu023 . 24526181 PMC4994837

[71] Weiland BJ, Heitzeg MM, Zald D, Cummiford C, Love T, Zucker RA, et al Relationship between impulsivity, prefrontal anticipatory activation, and striatal dopamine release during rewarded task performance. Psychiatry Res. 2014;223(3):244–52. 10.1016/j.pscychresns.2014.05.015 24969539 PMC4136473

[72] van den Bos W, Rodriguez CA, Schweitzer JB, McClure SM. Connectivity strength of dissociable striatal tracts predict individual differences in temporal discounting. J Neurosci. 2014;34(31):10298–310. 10.1523/JNEUROSCI.4105-13.2014 . 25080591 PMC4577570

[73] Castellanos-Ryan N, Struve M, Whelan R, Banaschewski T, Barker GJ, Bokde AL, et al. Neural and Cognitive Correlates of the Common and Specific Variance Across Externalizing Problems in Young Adolescence. Am J Psychiatry. 2014. 10.1176/appi.ajp.2014.13111499 . 25073448

[74] Critchley HD. Electrodermal responses: what happens in the brain. Neuroscientist. 2002;8(2):132–42. . 11954558 10.1177/107385840200800209

[75] Critchley HD. Psychophysiology of neural, cognitive and affective integration: fMRI and autonomic indicants. Int J Psychophysiol. 2009;73(2):88–94. 10.1016/j.ijpsycho.2009.01.012 19414044 PMC2722714

[76] Damasio AR. Descartes' Error: Emotion, Reason and the Human Brain. New York: Putnam; 1994.

[77] Dolan RJ. Emotion, cognition, and behavior. Science. 2002;298(5596):1191–4. . 12424363 10.1126/science.1076358

[78] Bechara A, Damasio H, Tranel D, Damasio AR. Deciding advantageously before knowing the advantageous strategy. Science. 1997;275(5304):1293–5. . 9036851 10.1126/science.275.5304.1293

[79] Frith CD, Allen HA. The skin conductance orienting response as an index of attention. Biol Psychol. 1983;17(1):27–39. . 6626635 10.1016/0301-0511(83)90064-9

[80] Delgado MR, Nearing KI, Ledoux JE, Phelps EA. Neural circuitry underlying the regulation of conditioned fear and its relation to extinction. Neuron. 2008;59(5):829–38. 10.1016/j.neuron.2008.06.029 18786365 PMC3061554

[81] Schiller D, Levy I, Niv Y, LeDoux JE, Phelps EA. From fear to safety and back: reversal of fear in the human brain. J Neurosci. 2008;28(45):11517–25. 10.1523/JNEUROSCI.2265-08.2008 18987188 PMC3844784

[82] Milad MR, Wright CI, Orr SP, Pitman RK, Quirk GJ, Rauch SL. Recall of fear extinction in humans activates the ventromedial prefrontal cortex and hippocampus in concert. Biol Psychiatry. 2007a;62(5):446–54. . 17217927 10.1016/j.biopsych.2006.10.011

[83] Critchley HD, Mathias CJ, Dolan RJ. Neural activity in the human brain relating to uncertainty and arousal during anticipation. Neuron. 2001;29(2):537–45. . 11239442 10.1016/s0896-6273(01)00225-2

[84] Fan J, Xu P, Van Dam NT, Eilam-Stock T, Gu X, Luo YJ, et al Spontaneous brain activity relates to autonomic arousal. J Neurosci. 2012;32(33):11176–86. 10.1523/JNEUROSCI.1172-12.2012 22895703 PMC3435430

[85] Nili U, Goldberg H, Weizman A, Dudai Y. Fear thou not: activity of frontal and temporal circuits in moments of real-life courage. Neuron. 2010;66(6):949–62. 10.1016/j.neuron.2010.06.009 20620879

[86] Sarlo M, Lotto L, Palomba D, Scozzari S, Rumiati R. Framing the ultimatum game: gender differences and autonomic responses. International journal of psychology: Journal international de psychologie. 2013;48(3):263–71. 10.1080/00207594.2012.656127 . 22494303

[87] Goldstein JM, Jerram M, Abbs B, Whitfield-Gabrieli S, Makris N. Sex differences in stress response circuitry activation dependent on female hormonal cycle. J Neurosci. 2010;30(2):431–8. 10.1523/JNEUROSCI.3021-09.2010 20071507 PMC2827936

[88] Litschauer B, Zauchner S, Huemer KH, Kafka-Lutzow A. Cardiovascular, endocrine, and receptor measures as related to sex and menstrual cycle phase. Psychosomatic medicine. 1998;60(2):219–26. . 9560873 10.1097/00006842-199803000-00019

[89] Fischer H, Fransson P, Wright CI, Backman L. Enhanced occipital and anterior cingulate activation in men but not in women during exposure to angry and fearful male faces. Cognitive, affective & behavioral neuroscience. 2004;4(3):326–34. . 15535168 10.3758/cabn.4.3.326

[90] Suarez EC, Saab PG, Llabre MM, Kuhn CM, Zimmerman E. Ethnicity, gender, and age effects on adrenoceptors and physiological responses to emotional stress. Psychophysiology. 2004;41(3):450–60. 10.1111/j.1469-8986.00161.x . 15102131

[91] Gomez P, Danuser B. Cardiovascular patterns associated with appetitive and defensive activation during affective picture viewing. Psychophysiology. 2010;47(3):540–9. 10.1111/j.1469-8986.2009.00953.x . 20030760

[92] Whiteside SP, Lynam DR. Understanding the role of impulsivity and externalizing psychopathology in alcohol abuse: application of the UPPS impulsive behavior scale. Experimental and clinical psychopharmacology. 2003;11(3):210–7. . 12940500 10.1037/1064-1297.11.3.210

[93] Torrubia R, Avila C, Molto J, Caseras X. The Sensitivity to Punishment and Sensitivity to Reward Questionnaire (SPSRQ) as a measure of Gray's anxiety and impulsivity dimensions. Personality and Individual Differences. 2001;31(6):837–62.

[94] Davis C, Fox J. Sensitivity to reward and body mass index (BMI): evidence for a non-linear relationship. Appetite. 2008;50(1):43–9. 10.1016/j.appet.2007.05.007 . 17614159

[95] Booth C, Hasking P. Social anxiety and alcohol consumption: the role of alcohol expectancies and reward sensitivity. Addictive behaviors. 2009;34(9):730–6. 10.1016/j.addbeh.2009.04.010 . 19464809

[96] Castella J, Perez J. Sensitivity to punishment and sensitivity to reward and traffic violations. Accident; analysis and prevention. 2004;36(6):947–52. 10.1016/j.aap.2003.10.003 . 15350871

[97] Leue A, Brocke B, Hoyer J. Reinforcement sensitivity of sex offenders and non-offenders: an experimental and psychometric study of reinforcement sensitivity theory. British journal of psychology. 2008;99(Pt 3):361–78. 10.1348/000712607X228519 . 17662171

[98] Lyvers M, Duff H, Basch V, Edwards MS. Rash impulsiveness and reward sensitivity in relation to risky drinking by university students: potential roles of frontal systems. Addictive behaviors. 2012;37(8):940–6. 10.1016/j.addbeh.2012.03.028 . 22521364

[99] Paquet C, Daniel M, Knauper B, Gauvin L, Kestens Y, Dube L. Interactive effects of reward sensitivity and residential fast-food restaurant exposure on fast-food consumption. The American journal of clinical nutrition. 2010;91(3):771–6. 10.3945/ajcn.2009.28648 . 20089726

[100] Rollins BY, Loken E, Savage JS, Birch LL. Measurement of food reinforcement in preschool children. Associations with food intake, BMI, and reward sensitivity. Appetite. 2014;72:21–7. 10.1016/j.appet.2013.09.018 . 24090537 PMC4262148

[101] Simons JS, Dvorak RD, Batien BD. Methamphetamine use in a rural college population: associations with marijuana use, sensitivity to punishment, and sensitivity to reward. Psychology of addictive behaviors: journal of the Society of Psychologists in Addictive Behaviors. 2008;22(3):444–9. 10.1037/0893-164X.22.3.444 . 18778139

[102] Li CS, Huang C, Lin W, Sun C. Gender differences in punishment and reward sensitivity in a Taiwanese sample of college students. Personality and Individual Differences. 2007;43(3):475–83.

[103] Constantinou E, Panayiotou G, Konstantinou N, Loutsiou-Ladd A, Kapardis A. Risky and aggressive driving in young adults: Personality matters. Accident; analysis and prevention. 2011;43(4):1323–31. 10.1016/j.aap.2011.02.002 . 21545861

[104] Scott-Parker B, Watson B, King MJ, Hyde MK. The influence of sensitivity to reward and punishment, propensity for sensation seeking, depression, and anxiety on the risky behaviour of novice drivers: a path model. British journal of psychology. 2012;103(2):248–67. 10.1111/j.2044-8295.2011.02069.x . 22506749

[105] Scott-Parker B, Watson B, King MJ, Hyde MK. A further exploration of sensation seeking propensity, reward sensitivity, depression, anxiety, and the risky behaviour of young novice drivers in a structural equation model. Accident; analysis and prevention. 2013;50:465–71. 10.1016/j.aap.2012.05.027 . 22770376

[106] White TL, Lejuez CW, de Wit H. Personality and gender differences in effects of d-amphetamine on risk taking. Experimental and clinical psychopharmacology. 2007;15(6):599–609. 10.1037/1064-1297.15.6.599 . 18179313

[107] Perkins KA, Lerman C, Coddington SB, Jetton C, Karelitz JL, Scott JA, et al Initial nicotine sensitivity in humans as a function of impulsivity. Psychopharmacology. 2008;200(4):529–44. 10.1007/s00213-008-1231-7 . 18604520

[108] Lombardo MV, Ashwin E, Auyeung B, Chakrabarti B, Lai MC, Taylor K, et al Fetal programming effects of testosterone on the reward system and behavioral approach tendencies in humans. Biol Psychiatry. 2012;72(10):839–47. 10.1016/j.biopsych.2012.05.027 22763187 PMC3485553

[109] Cservenka A, Herting MM, Seghete KL, Hudson KA, Nagel BJ. High and low sensation seeking adolescents show distinct patterns of brain activity during reward processing. Neuroimage. 2013;66:184–93. 10.1016/j.neuroimage.2012.11.003 23142276 PMC3604176

[110] Li Y, Qiao L, Sun J, Wei D, Li W, Qiu J, et al Gender-specific neuroanatomical basis of behavioral inhibition/approach systems (BIS/BAS) in a large sample of young adults: a voxel-based morphometric investigation. Behav Brain Res. 2014;274:400–8. 10.1016/j.bbr.2014.08.041 . 25172180

[111] Urosevic S, Collins P, Muetzel R, Lim K, Luciana M. Longitudinal changes in behavioral approach system sensitivity and brain structures involved in reward processing during adolescence. Developmental psychology. 2012;48(5):1488–500. 10.1037/a0027502 22390662 PMC3370133

[112] Lijffijt M, Bekker EM, Quik EH, Bakker J, Kenemans JL, Verbaten MN. Differences between low and high trait impulsivity are not associated with differences in inhibitory motor control. Journal of attention disorders. 2004;8(1):25–32. . 15669600 10.1177/108705470400800104

[113] Wilbertz T, Deserno L, Horstmann A, Neumann J, Villringer A, Heinze HJ, et al Response inhibition and its relation to multidimensional impulsivity. Neuroimage. 2014;103:241–8. 10.1016/j.neuroimage.2014.09.021 . 25241087

